# Critical roles of CTP synthase N-terminal in cytoophidium assembly

**DOI:** 10.1016/j.yexcr.2017.03.042

**Published:** 2017-05-15

**Authors:** Yong Huang, Jin-Jun Wang, Sanjay Ghosh, Ji-Long Liu

**Affiliations:** a MRC Functional Genomics Unit, Department of Physiology, Anatomy and Genetics, University of Oxford, Oxford OX1 3PT, United Kingdom; b Key Laboratory of Entomology and Pest Control Engineering, College of Plant Protection, Southwest University, Chongqing, China; c School of Life Science and Technology, ShanghaiTech University, Shanghai 201210, China

**Keywords:** CTP synthase, Cytoophidium, Intracellular filaments, *Drosophila*, Metabolic cell biology, Membrane-less organelle

## Abstract

Several metabolic enzymes assemble into distinct intracellular structures in prokaryotes and eukaryotes suggesting an important functional role in cell physiology. The CTP-generating enzyme CTP synthase forms long filamentous structures termed cytoophidia in bacteria, yeast, fruit flies and human cells independent of its catalytic activity. However, the amino acid determinants for protein-protein interaction necessary for polymerisation remained unknown. In this study, we systematically analysed the role of the conserved N-terminal of *Drosophila* CTP synthase in cytoophidium assembly. Our mutational analyses identified three key amino acid residues within this region that play an instructive role in organisation of CTP synthase into a filamentous structure. Co-transfection assays demonstrated formation of heteromeric CTP synthase filaments which is disrupted by protein carrying a mutated N-terminal alanine residue thus revealing a dominant-negative activity. Interestingly, the dominant-negative activity is supressed by the CTP synthase inhibitor DON. Furthermore, we found that the amino acids at the corresponding position in the human protein exhibit similar properties suggesting conservation of their function through evolution. Our data suggest that cytoophidium assembly is a multi-step process involving N-terminal-dependent sequential interactions between correctly folded structural units and provide insights into the assembly of these enigmatic structures.

## Highlights

• CTP synthase mutational analyses reveal N-terminal amino acids that regulate filament self-assembly.
• Amino acid 20 of CTP synthase plays key role in protein interactions necessary for polymerisation.
• The dominant-negative activity is supressed by CTP synthase inhibitor DON.
• The functional properties of the amino acids are conserved in Drosophila and human CTP synthases.

## 1 Introduction

Intracellular compartmentation of biological processes allows a higher level of complexity in eukaryotic cells. In addition to the canonical membrane-bound organelles, several membrane-less macromolecular assemblies consisting of RNA and proteins have been discovered [1], [2], [3], [4]. Such structures are dynamic and responsive to both intrinsic and extrinsic cues suggesting a functional role in cellular response and adaptation. Interestingly, several metabolic enzymes self-assemble into structurally diverse higher order structures in the cytoplasm [5], [6], [7], [8] indicating an important role in metabolic programming. Such organisational specificity might maximise metabolic efficiency and enable reversible storage of the enzymes. However, the underlying principles that govern the formation, maintenance and regulation of these structures remain poorly understood.

CTP synthase is an essential enzyme that catalyses the *de novo* formation of CTP from UTP and ATP using glutamate as nitrogen source [9]. The enzyme consists of an N-terminal synthetase/amidoligase (ALase) domain that binds ATP and UTP, a helical interdomain linker and a C-terminal glutamine amino-transferase (GATase) domain [10], [11]. At physiological concentrations, binding of the substrates ATP and UTP induces oligomerisation of the inactive dimers to form enzymatically active tetramers such that the ALase active site is formed of three different subunits while a tubular vestibule connects the GATase active site and the GATase/ALase interface [10]. Glutamine hydrolysis occurs in the GATase domain and the resulting ammonia is subsequently transferred *via* the tunnel to the N-terminal synthetase domain where it reacts with the phosphorylated UTP to form CTP [10], [12], [13]. Interestingly, the product CTP acts as a competitive inhibitor which is essential to maintain proper intracellular CTP levels [14], [15], [16]. These biochemical properties are conserved in bacterial, yeast and mammalian enzymes. Since CTP is a key precursor in the synthesis of nucleic acids and membrane phospholipids, CTP synthase has been an attractive target for the development of anti-cancer, anti-viral and anti-protozoal agents [17], [18], [19], [20].

Recently, CTP synthase was found to organise into long filamentous structures in bacteria, yeast, *Drosophila* and human cells [6], [21], [22], [23], [24], for reviews see [25], [26], [27], suggesting that polymerisation is an evolutionary conserved property of this enzyme. While the bacterial protein self-assembles into filaments *in vitro* and *in vivo* and interacts with intermediate filaments to determine cell shape [21], [28], the frequency of filamentation in yeast is influenced by the availability of nutrients and the growth phase [6], [29]. In *Drosophila* the CTP synthase polymeric structures (called cytoophidia) are found in several tissues including the germ line [6], [22], [24], [30], [31], [32], [33]. Strikingly, CTP synthase filaments have been found both in the nucleus and cytoplasm in yeast and humans [34], [35]. However, structural organisation of the CTP synthase filaments might differ between organisms. While the filaments are composed of inactive tetramers in bacteria, they may be formed of inactive monomers and/or dimers in yeast and *Drosophila* [28], [32], [36]. In addition, binding of the CTP synthase inhibitor DON (6-Diazo-5-oxo-L-norleucine) disrupts the linear structures in bacteria while it promotes assembly in *Drosophila* and humans [24], [28]. Finally, mutation of the DON-binding site (C388G) significantly reduced linear CTP synthase structures in bacteria while a similar effect was observed with the CTP-binding site (E160K) mutant in yeast and *Drosophila* [21], [32], [36] suggesting a close link between enzyme activity and filament-forming capability. However, the role of amino acid residues outside of the enzyme active site has not been investigated.

Here, we report structure-function analysis of N-terminal of *Drosophila* CTP synthase which reveals key amino acid residues that regulate distinct steps in the formation of higher order structures necessary for assembly of the filamentous cytoophidium.

## 2 Results

### 2.1 *Drosophila* CTPsyn forms cytoophidia in clone 8 cells

**Fig. 1:**
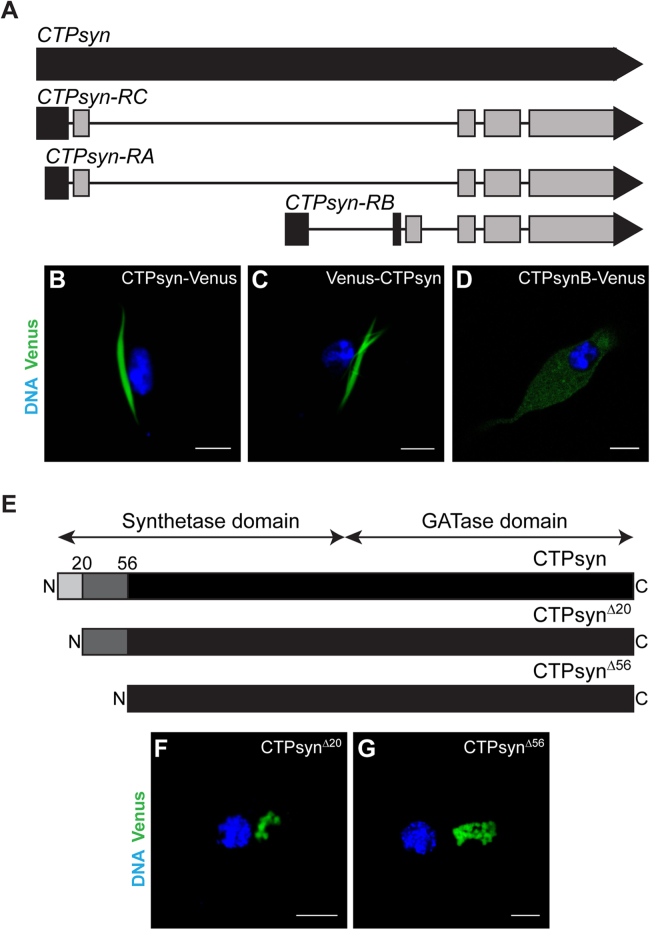
**The N-terminal of*****Drosophila*****CTP synthase is required for cytoophidium formation in clone 8 cells.** (A) Schematic diagram showing genomic organisation of *Drosophila* CTP synthase gene (*CTPsyn*). Three transcript isoforms *CTPsyn-RA*, *CTPsyn-RB* and *CTPsyn-RC* originate from the locus and differ in their 5'-UTR and first exon sequences. The grey boxes and interconnecting lines represent the exons and introns, respectively, while the 5′- and 3′-UTR sequences are shown in black boxes and triangles. (B-D) Distribution patterns of Venus-tagged CTPsyn isoforms expressed in clone 8 cells. CTPsyn fused with the fluorescent protein Venus at the C-terminus (CTPsyn-Venus, B**)** and N-terminus (Venus-CTPsyn, C) assembles into a filamentous cytoophidium while the CTPsynB isoform tagged at the C-terminus with Venus (CTPsynB-Venus, D) shows cytoplasmic distribution. (E) Diagram showing CTPsyn deletion constructs CTPsyn^Δ20^ and CTPsyn^Δ56^ with 20 and 56 amino acids eliminated from the N-terminus, respectively. These truncated proteins organise into large cytoplasmic clusters (CTPsyn^Δ20^, F and CTPsyn^Δ56^, G). DNA stained with Hoechst is shown in blue while the Venus-tagged proteins are in green. GATase=Glutamine amino-transferase. Scale bar=5 µm.

The gene model for *Drosophila CTPsyn* shows three transcripts associated with the locus (flybase.org). The transcripts *CTPsyn-RA* and *CTPsyn-RC* encode identical polypeptides but differ in the length of their 5’UTRs (Fig. 1**A**). This protein is hereafter referred to as CTPsyn. Transgenic expression of this protein (CTPsyn-Venus) in *Drosophila* rescues the *CTPsyn* mutant phenotype [33] and results in filament assembly in tissues lacking endogenous cytoophidia [31] indicating self-assembly of the functional protein. Furthermore, the overexpressed protein co-localises with endogenous CTPsyn filaments. In contrast, CTPsynB (encoded by the *CTPsyn-RB* transcript, Fig. 1**A**) shows cytoplasmic distribution and does not assemble into cytoophidia [31]. CTPsynB is identical to CTPsyn except at the N-terminal end - the first 52 amino acid residues of CTPsynB protein differ considerably from the corresponding 56 amino acids of CTPsyn (Fig. S1A). Indeed, the 56 amino acids at the N-terminal of CTPsyn are required for polymerisation as their deletion disrupts filament assembly *in vivo* [31]. This argues for an essential role of the unique N-terminal of CTPsyn in cytoophidium formation. Therefore, we investigated the role of CTPsyn N-terminal amino acid residues in filament assembly.

To determine the functional role of the CTPsyn N-terminal in the formation of a filamentous structure, we performed transient transfection-based assays using *Drosophila* clone 8 cells. In this cell line the endogenous CTPsyn protein assembles into a filamentous structure at low frequency (less than 10% cells) only in presence of the CTP synthase inhibitor DON (Fig. S2). Therefore, for direct visualisation within cells and monitor intracellular distribution, CTPsyn was tagged at its C-terminal end with the fluorescent protein Venus (CTPsyn-Venus). As shown in Fig. 1**B**, CTPsyn-Venus assembled into a linear filament-like structure in clone 8 cells as observed previously in *Drosophila* tissues [31], [32], [33]. We observed a single cytoophidium per cell (average length ~10 µm, Fig. 4**D**) and failed to detect any signal in the cytoplasm suggesting that the majority of the protein accumulates in the filaments. Similarly, Venus-CTPsyn (tag at the N-terminus) also formed cytoophidia in the cytoplasm (Fig. 1**C,** [31]). CTPsynB-Venus, in contrast, did not assemble into a polymeric structure – in most cells the fluorescent signal was distributed uniformly in the cytoplasm (Fig. 1**D**, [31]). Thus, the CTPsyn-specific filament-forming property as reported in *Drosophila in vivo* is reproducible in the clone 8 cells.

### 2.2 **N-terminus amino acids are necessary for CTPsyn filamentation in*****Drosophila***

Sequence alignment reveals that the 56 amino acids at the N-terminus of CTPsyn differ considerably from the corresponding region of CTPsynB (Fig. S1A). However, this region shows significant identity in CTPsyn orthologues across the phyla particularly in regions containing glycine residues (Fig. S1B). To test if the conserved regions within the N-terminal are required for filament assembly, we divided the 56 amino acid stretch into two parts (aa 1–20 and aa 21–56) and generated CTPsyn-Venus constructs wherein either aa 2–20 (CTPsyn^Δ2^^0^) or aa 2–56 (CTPsyn^Δ56^) were deleted (Fig. 1**E**). As shown in Fig. 1**F**, deletion of the N-terminal 20 amino acids alone is sufficient to disrupt cytoophidium assembly; the CTPsyn^Δ2^^0^ protein formed distinct cytoplasmic structures which appear as large clusters of signal (upto 2 µm). A similar distribution of the fluorescent signal is observed for the CTPsyn^Δ56^ protein (Fig. 1**G**). This result indicates that CTPsyn amino acid residues 21–56 and the rest of the protein are not sufficient for filament assembly, rather the first 20 amino acids of the polypeptide are required for its organisation into a cytoophidium structure. Additionally, lack of a uniform cytoplasmic signal from these proteins (as observed for CTPsynB-Venus) suggests a defect in later steps of the polymerisation process following sequestration. These clusters might represent an intermediate in filament assembly (see below). Notably, the yeast CTP synthase (Ura7) assembles into filaments (~70%) and foci-like structures (~30%) in the cytoplasm, and the frequency of formation of these structures is determined by certain amino acid residues as well as environmental stress conditions [36].

Previous studies have identified multiple amino acids in CTP synthase that affect its filament assembly and morphology [21], [28], [32], [36]. However, the role of N-terminal residues in this process has not been investigated. Notably, structural analyses show that the N-terminal domain is involved in contact between CTP synthase monomers for tetramer assembly [10]. Therefore, we focussed our analyses on the first 20 amino acids of CTPsyn for their role in cytoophidium formation. This region contains several amino acids, particularly glycine residues, which are conserved across eukaryotes and prokaryotes (Fig. S1B). Many nucleotide-binding domains are rich in glycine moieties that either bind to nucleotides directly and/or provide a structural role [37], [38], [39]. The crystal structure of *E.coli* CTP synthase protein (hereafter called pyrG) revealed that the amino acid residues 15–20 constitute a P-loop-like structure that binds the adenine nucleotide – this provides a structural basis for ATP-dependent tetramerisation of the enzyme [40]. Structural analyses of the human CTPS1 and *Sulfolobus solfataricus* CTP synthase revealed a similar feature involving the corresponding amino acid residues [41], [42]. Furthermore, CTPsyn amino acids 1–20 could be targets of multiple post-translation modifications. Indeed, such modifications (phosphorylation and ubiquitination) has been shown to regulate enzyme activity and filament assembly across species [43], [44], [45], [46], [47], [48], [49], [50]. Recently, *myc* has been shown to regulate cytoophidium assembly in *Drosophila* [51].

**Fig. 2:**
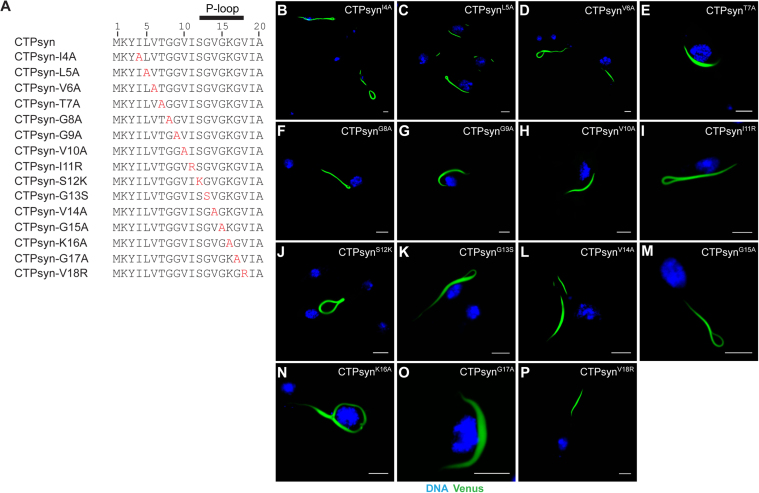
**Mutational analysis of*****Drosophila*****CTPsyn N-terminal amino acid residues.** (A) The N-terminal 1–20 amino acid sequence of *Drosophila* CTPsyn and the constructs carrying mutations targeting amino acids 4–18 (shown in red). The amino acids 12–18 that constitute the putative P-loop-like structure are indicated at the top of the sequence with a solid line. (B–P) Distribution of the mutant CTPsyn proteins derived from the constructs shown in (A) which are tagged at the C-terminus with Venus protein. All mutant proteins form cytoophidia similar to the wild-type CTPsyn. DNA staining with Hoescht is shown in blue and the Venus-tagged proteins are in green. Scale bar=5 µm.

To identify the amino acid(s) within the 1–20 region that determine cytoophidium assembly, we systematically mutated individual amino acids in the CTPsyn-Venus protein (Fig. 2) and monitored their distribution within cells. As shown in Fig. 2**B-P**, expression of proteins with mutations targeting aa 4–18 did not affect cytoophidium formation – the mutant proteins consistently formed filaments similar to that observed for the wild-type protein. This demonstrates that the CTPsyn amino acid residues constituting the putative P-loop-like structure in *Drosophila* (aa 12–18) are dispensable for filament assembly. Additionally, the glycine residues in this region do not appear to play any structural role in cytoophidium formation.

### 2.3 **Heterologous N-termini from human and yeast CTP synthase support*****Drosophila*****CTPsyn filamentation**

**Fig. 3:**
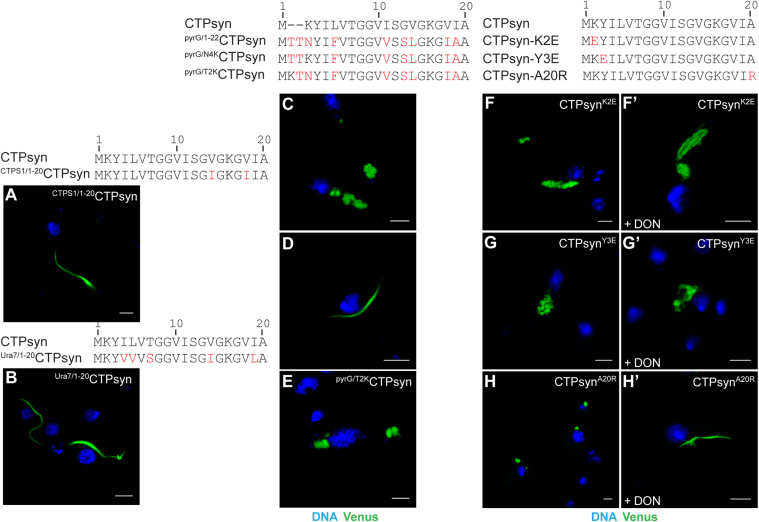
**The conserved amino acid residues K2, Y3 and A20 at the CTP synthase N-terminus are necessary for filament assembly.** Replacement of N-terminal 20 amino acids of the *Drosophila* CTPsyn with the corresponding human (^CTPS1/1–20^CTPsyn, A), *S. cerevisiae* (^Ura7/1–20^CTPsyn, B) and *E. coli* (^pyrG/1–22^CTPsyn, C) CTP synthase peptide shows that the bacterial sequence does not support cytoophidium formation. However, mutating the amino acid at position 4 (^pyrG/N4K^CTPsyn, D) but not position 2 (^pyrG/N4K^CTPsyn, E) to lysine residue (K) induces filament formation. The amino acid sequences (1–20) of the proteins are shown at the top of each panel with the amino acids different from the *Drosophila* CTPsyn marked in red. (F-H) Mutation of the *Drosophila* CTPsyn amino acids at position 2 (K2E), position 3 (Y3E) and position 20 (A20R) disrupts cytoophidium formation. CTPsyn^K2E^ and CTPsyn^Y3E^ proteins form large clusters (F and G, respectively) while CTPsyn^A20R^ assembles into smaller cytoplasmic foci-like structures (H). In the presence of the CTPsyn inhibitor DON (F'-H') only CTPsyn^A20R^ protein assembles into a cytoophidium (H′). DNA stained with Hoechst is shown in blue while the Venus-tagged proteins are in green. Scale bar=5 µm.

Comparison of the CTP synthase N-terminal sequences (aa 1–20) from bacteria to humans shows remarkable conservation of several amino acid residues indicating their essential function in enzyme activity and/or regulation (Fig. S1B). Previous studies have shown that CTP synthase isoforms in human (CTPS1 and CTPS2) and budding yeast (Ura7 and Ura8) assemble into cytoophidia [6], [29], [34]. Notably, the human and yeast CTP synthase isoforms have identical 20 amino acids at their N-terminus which show significant identity with the corresponding region of *Drosophila* CTPsyn (CTPS1 *vs* CTPsyn 90%, Ura7 *vs* CTPsyn 75%, Fig. 3**A, B**). Thus we tested if heterologous N-termini from human and yeast CTP synthase could support *Drosophila* CTPsyn cytoophidium assembly. Towards this, we performed N-terminal swapping experiments wherein aa 1–20 of *Drosophila* CTPsyn-Venus was replaced with aa 1–20 from Ura7 and CTPS1 thus generating the constructs ^Ura7/1–20^CTPsyn and ^CTPS1/1–20^CTPsyn, respectively. As shown in Fig. 3**A, B**, N-terminal amino acid residues 1–20 from human and yeast CTP synthase did not affect CTPsyn cytoophidium assembly in *Drosophila* cells. This result validates our mutational analyses and further shows that replacement of the isoleucine residue (I) at position 19 in CTPsyn by leucine (L) does not affect polymerisation (as illustrated by ^Ura7/1–20^CTPsyn protein). Taken together, we conclude that the identities of the CTPsyn aa 4–19 are non-essential for cytoophidium formation.

### 2.4 Three amino acid residues at the CTPsyn N-terminus are critical for cytoophidium assembly

Mutation of CTPsyn N-terminal amino acids at position 2 (CTPsyn^K2E^), 3 (CTPsyn^Y3E^) and 20 (CTPsyn^A20R^), however, disrupted cytoophidium formation (Fig. 3**F-H**) highlighting the essential role of these residues in proper assembly of the filaments. Instead of forming long filaments, the mutant proteins accumulated in morphologically distinct structures in the cytoplasm. For example, in contrast with CTPsyn, the CTPsyn^A20R^ protein is found in a punctate foci-like structure within cells (Fig. 3**H**) while the fluorescent signal in CTPsyn^K2E^ and CTPsyn^Y3E^ expressing cells is sequestered into larger aggregates in the cytoplasm (Fig. 3**F, G**) similar to that observed for the CTPsyn^Δ20^ protein (Fig. 1**F**). Notably, mutations in the CTP-binding site of CTP synthase (E165K) resulted in the loss of filamentous structure and increased formation of punctate structures in yeast and *Drosophila* [32], [36]. Quantitative analysis, however, revealed a differential effect of the mutations on the filament-forming capability of CTPsyn. The vast majority of cells expressing A20R and Y3E mutant proteins lacked filamentous structures (92% and 90% respectively) while K2E mutation reduced the frequency of cytoophidium formation to 60% (Fig. S1C). These variable effects suggest separate roles of these amino acids in polymeric assembly and point towards an interaction *in cis* that is necessary for filamentation. Since tyrosine (Y) residue is a potential phosphorylation target by protein kinases and cytoophidium formation is severely compromised by Y3E mutation, we speculate that this amino acid might be phosphorylated which could regulate filament assembly. Indeed CTP synthase is a target for protein kinases and undergoes phosphorylation [43], [44], [45], [46], [47], [48]. Notably, the amino acid residues at position 2 (K), 3 (Y) and 20 (A) of CTP synthase are conserved in human, yeast and *Drosophila* (Fig. S1B).

### 2.5 The spatial relationship among key amino acid residues is crucial for CTPsyn filamentation

Sequence alignment shows that *E.coli* pyrG N-terminal is 2 amino acids longer and displays lower identity (65%) with CTPsyn. In addition, the N-terminus of the polypeptide lacks the conserved lysine residue at position 2 (Fig. 3 and S1B). To test if the pyrG N-terminal can support *Drosophila* CTPsyn cytoophidium assembly, we replaced CTPsyn aa 1–20 with pyrG aa 1–22 (^pyrG/1–22^CTPsyn) and expressed the chimeric protein in *Drosophila* cells. Interestingly, ^pyrG/1–22^CTPsyn failed to organise into a filamentous structure in most cells (80%, Fig. S1C) and accumulated into large aggregates (Fig. 3**C**) similar to the structures observed with CTPsyn^Δ20^, CTPsyn^K2E^ and CTPsyn^Y3E^ proteins. This further demonstrates the importance of the lysine residue at position 2 in *Drosophila* CTPsyn in cytoophidium assembly. However, the failure to form filaments is intriguing as pyrG assembles into a filamentous structure *in vitro* and *in vivo* in prokaryotes [21]. The observed inability of ^pyrG/1–22^CTPsyn to organise into cytoophidia in *Drosophila* could be due to altered distance of the N-terminal end from conserved amino acids elsewhere in CTPsyn and/or the lack of a conserved lysine (K) residue at position 2 or 4. To address this, we mutated the asparagine residue to lysine (N4K) in the ^pyrG/1–22^CTPsyn construct to generate ^pyrG/N4K^CTPsyn. Remarkably, the ^pyrG/N4K^CTPsyn protein formed long filaments similar to CTPsyn cytoophidia in all cells expressing the protein (Fig. 3**D**, Fig. S1C). To test if the position of the lysine residue relative to the N-terminal end and/or conserved tyrosine (Y) residue is essential for filament formation, we generated an additional construct ^pyrG/T2K^CTPsyn wherein the threonine at position 2 in ^pyrG/1–22^CTPsyn was mutated to lysine (T2K). This protein, however, failed to form cytoophidia and was partitioned into aggregates in the cytoplasm similar to ^pyrG/1–22^CTPsyn (Fig. 3**E**). Thus, we conclude that the proximity of the lysine residue (K) to the conserved tyrosine (Y) at positions 2 and 3 of CTPsyn is essential for cytoophidium formation and that its distance from the N-terminal end is not a factor for polymerisation. Furthermore, as observed before, this experiment highlights the dispensability of the isoleucine residue (I) at position 19 for filamentation as the ^pyrG/N4K^CTPsyn protein contains an alanine residue (A) at the corresponding position (Fig. 3). Our data further suggest that species-specific cross-talk between the N-terminal and the rest of protein likely determines the conformation necessary for organisation into a cytoophidium.

### 2.6 **CTPsyn**^**A20R**^**foci form filaments in presence of a glutamine analogue**

We next investigated whether the CTPsyn^A20R^ foci and the aggregate-like structure formed by CTPsyn^K2E^ and CTPsyn^Y3E^ proteins are dynamic structures and responsive to the CTPsyn inhibitor DON (6-Diazo-5-oxo-L-norleucine). DON is a glutamine analogue that covalently binds the glutaminase active site in the amidotransferase (GATase) domain of CTPsyn, thus irreversibly inactivating the enzyme activity [52]. An earlier study suggested that DON may induce structural alterations in CTPsyn that may lead to polymerisation [28]. Indeed, in *Drosophila* and human cells DON promotes cytoophidium assembly [24] which argues for the presence of catalytically inactive enzyme in the filaments. To determine the effect of DON on the cytoplasmic structures formed by the mutant proteins, we incubated the transfected cells with DON and monitored the distribution of the fluorescent signal. CTPsyn^A20R^ protein organised into filamentous structures similar to the wild-type cytoophidia and we failed to detect any cytoplasmic foci (Fig. 3**H′**). This effect was specific to CTPsyn^A20R^ protein as the distribution of CTPsyn^K2E^ and CTPsyn^Y3E^ remained unchanged in presence of DON (Fig. 3**F′, G′**). Therefore, binding of DON to the GATase domain of CTPsyn^A20R^ may induce a conformational change that masks the deleterious effect of the mutation on filament assembly. This shows that CTPsyn^A20R^ foci are reversible higher order structures that are responsive to regulation by glutamine raising the possibility that these foci are not inert bodies but rather they represent an intermediate in cytoophidium assembly. Since inactive CTP synthase enzyme has been shown to polymerise into cytoophidia [28], [32], [36], it is possible that the alanine residue at position 20 of CTPsyn might regulate enzyme activity and consequently filament formation.

### 2.7 The A20 residue of human CTP synthase is critical for filamentation

**Fig. 4:**
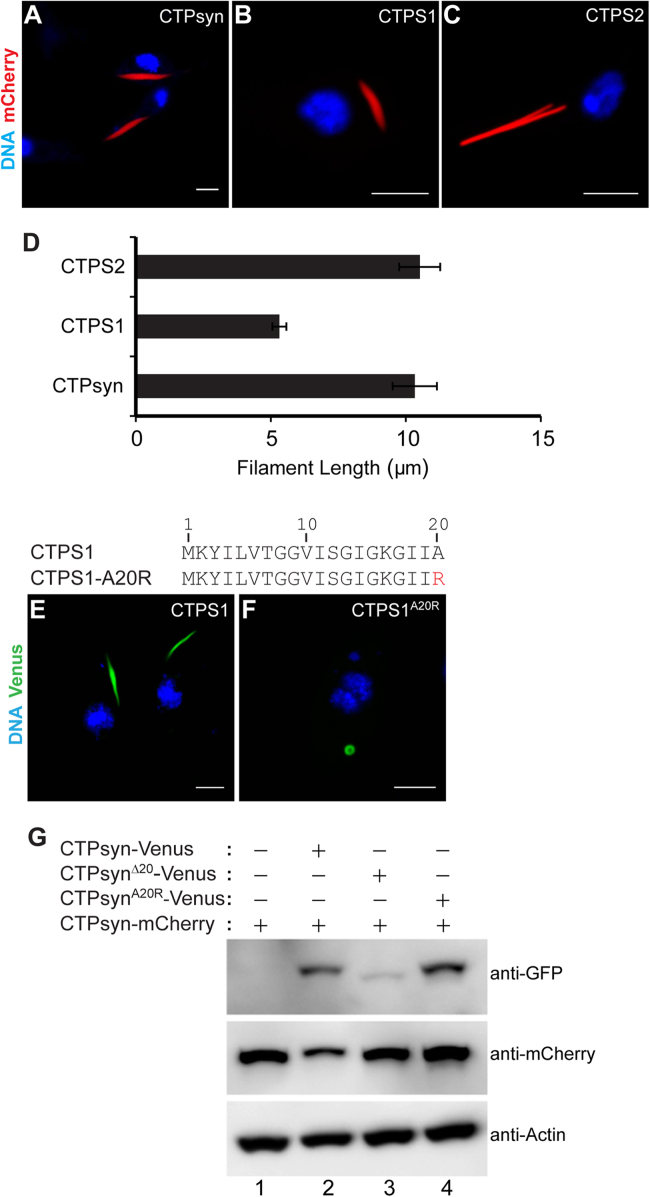
**Expression of*****Drosophila*****and human CTP synthase proteins.** Expression of mCherry-tagged *Drosophila* CTPsyn (A) as well as human CTPS1 (B) and CTPS2 (C) in clone 8 cells shows cytoophidium formation. (D) Quantitative comparison of the filament length of *Drosophila* and human cytoophidia shows that CTPS1 filaments are shorter (~50%) than CTPsyn and CTPS2 filaments. The error bar show mean±s.e.m. At least 75 transgenic cells were counted in each case. Expression of Venus-tagged human CTP synthase (CTPS1, E) in clone 8 cells results in formation of filamentous cytoophidia while the A20R mutant protein (CTPS1^A20R^, F) forms cytoplasmic foci similar to the corresponding *Drosophila* protein. The amino acid sequence (1–20) of CTPS1 and the mutant construct is shown at the top of the panels with the mutated amino acid in red. DNA is shown in blue while the Venus-tagged proteins are in green. Scale bar=5 µm. (G) Western blot using extracts from cells co-expressing Venus- and mCherry-tagged CTPsyn proteins. The primary antibody used to stain the blot is shown on the right side of the panel.

Next, we examined whether the alanine residue (A20) in human CTP synthase could have a similar role in cytoophidium formation. In humans, there are two isoforms of CTP synthase (CTPS1 and CTPS2) which when expressed in the mammalian cell lines polymerise into cytoophidia [24], [34]. However, the distribution of ectopically expressed human CTP synthase in *Drosophila* cells is unknown. To determine if CTPS1 and CTPS2 could assemble into cytoplasmic structures, we transfected clone 8 cells with mCherry-tagged constructs and monitored their distribution. Both CTPS1 and CTPS2 formed filaments similar to *Drosophila* CTPsyn (Fig. 4**A-C**). Quantification of the filament length showed that CTPS1 filaments were consistently shorter (~50%) as compared with CTPS2 and CTPsyn cytoophidia (Fig. 4**D**). Similar observations were made with the CTPS1- and CTPS2-Venus proteins. This analysis demonstrates that human CTP synthase enzyme can self-assemble and that the co-factors required for polymerisation are conserved in *Drosophila* and humans. Expression of CTPS1^A20R^, however, led to the formation of cytoplasmic foci similar to CTPsyn^A20R^ protein in all cells expressing the human protein (Fig. 4**F**, Fig. S1C). This result demonstrates that the N-terminal alanine residue at position 20 in CTP synthase polypeptide is critical for polymerisation and its functional role is conserved across evolution.

### 2.8 N-terminal dependent protein interactions are necessary for cytoophidium assembly

**Fig. 5:**
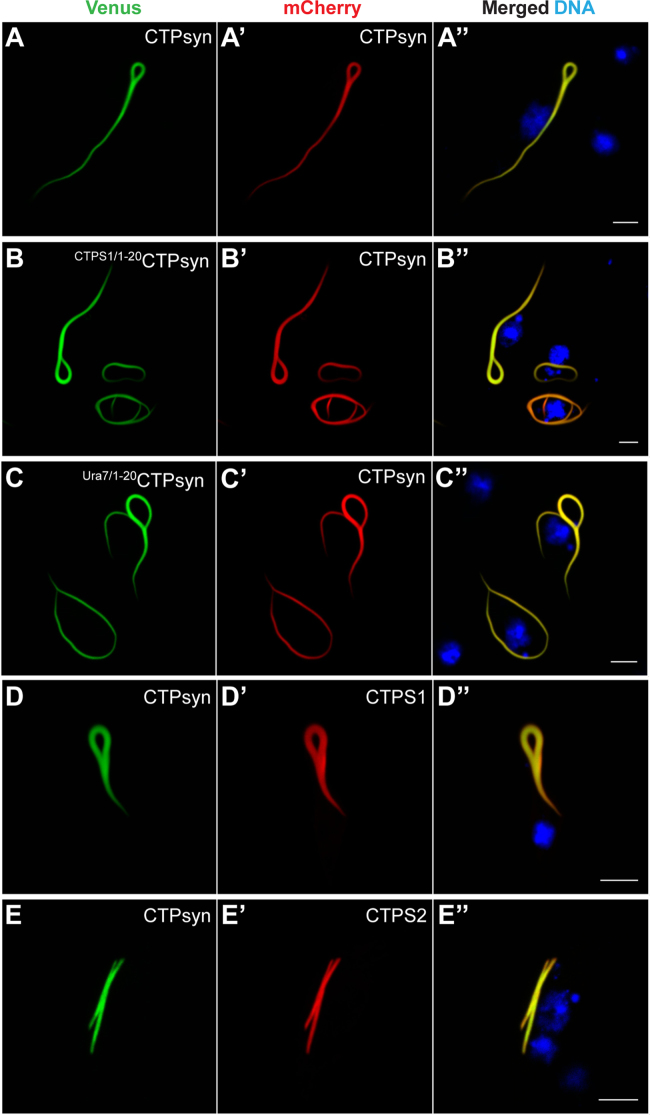
**Formation of heteromeric cytoophidia containing*****Drosophila*****and human CTP synthase proteins.** Co-expression of mCherry-tagged *Drosophila* CTPsyn (A′, B′ and C′) and Venus-tagged CTP synthase proteins (CTPsyn, A, ^CTPS1/1−20^CTPsyn, B and ^Ura7/1−20^CTPsyn, C) results in significant overlap of the signals (A′′, B′′, C′′) indicating co-assembly into the same cytoophidium. A similar result is obtained when epitope-tagged *Drosophila* (CTPsyn-Venus, D, E) and human (CTPS1-mCherry, D′, CTPS2-mCherry, E’) proteins are expressed together demonstrating formation of heteromeric structures (D′′, E′′). DNA staining with Hoescht is shown in blue and the Venus- and mCherry-tagged proteins are in green and red, respectively. Scale bar=5 µm.

**Fig. 6:**
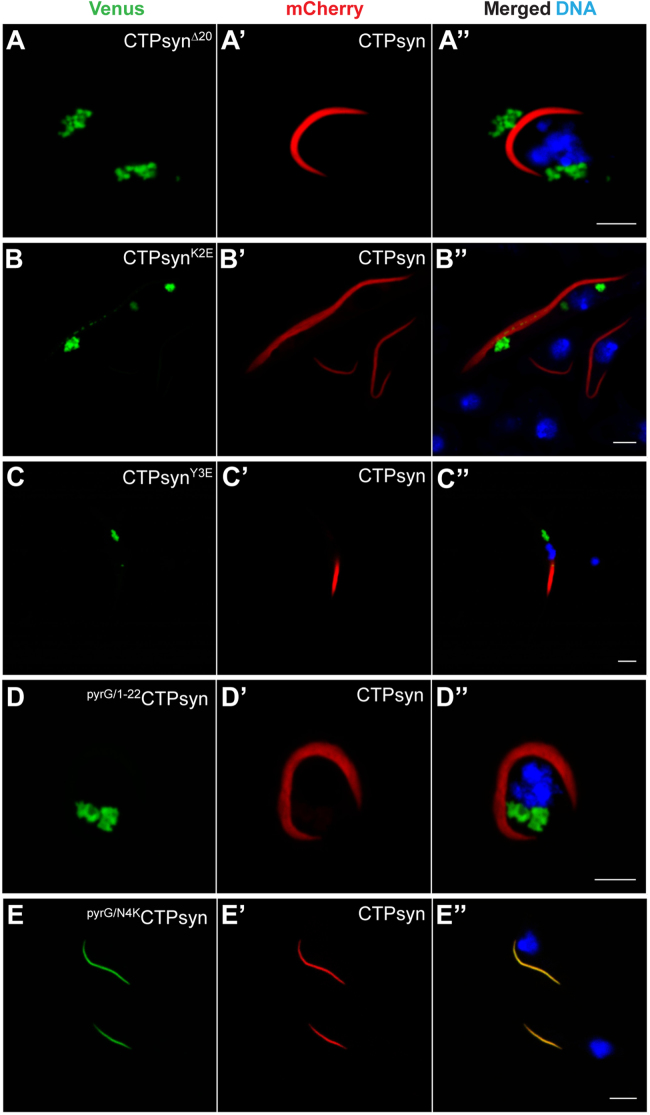
**Distribution of the*****Drosophila*****CTPsyn wild-type and mutant proteins in cells co-expressing the constructs.** Wild-type CTPsyn-mCherry (A′-E′) was transfected along with Venus-tagged CTPsyn mutant proteins CTPsyn^Δ20^ (A), CTPsyn^Κ2Ε^ (B), CTPsyn^Y3E^ (C), ^pyrG/1−22^CTPsyn (D) and ^pyrG/N4K^CTPsyn (E), and their distribution in the cytoplasm monitored. While CTPsyn^Δ20^, CTPsyn^Κ2Ε^ , CTPsyn^Y3E^ and ^pyrG/1−22^CTPsyn proteins assembled into aggregates that did not overlap with CTPsyn cytoophidia (A′′-D′′), ^pyrG/N4K^CTPsyn co-assembled with the cytoophidia (E′′). DNA staining with Hoescht is shown in blue and the Venus- and mCherry-tagged proteins are in green and red, respectively. Scale bar=5 µm.

The CTP synthase monomer and dimers are catalytically inactive. However, in presence of nucleotides (ATP, UTP and GTP) the protein becomes enzymatically active following tetramer assembly. Mutations in the dimer/tetramer interface have been shown to affect cytoophidium assembly [32], [36] underlining its importance in the regulation of enzyme activity and filament formation. Interestingly, structural analyses show that the CTPS N-terminal region lies at the tetramerization interface and our results uncovered essential roles of three amino acids in this region for self-assembly into filaments. We therefore investigated if the CTPsyn N-terminal 1–20 amino acids are involved in protein-protein interactions necessary for higher order assembly into cytoophidium structure. To address this, we generated CTP synthase constructs tagged at the C-terminus with the red fluorescent protein mCherry (Fig. 4**A-C**) and performed co-transfection assays along with the Venus-tagged constructs. As shown in Fig. 5**A-A′′**, CTPsyn-Venus co-localised fully with CTPsyn-mCherry demonstrating assembly into the same cytoophidium structure; both proteins are expressed at comparable levels (Fig. 4**G**, lane 2**)**. A similar result was obtained when CTPsyn-mCherry was expressed with the chimeric proteins ^CTPS1/1–20^CTPsyn and ^Ura7/1–20^CTPsyn (Fig. 5**B-B′′**, **C-C′′**). This strongly suggests that aa 4–19 are not involved in interactions necessary for recruitment of proteins into the filamentous structure. To determine if CTP synthase homologues have the capacity to associate with *Drosophila* CTPsyn cytoophidia, we co-expressed CTPsyn together with human CTPS1 and CTPS2. Indeed, both human proteins co-localised fully with CTPsyn showing assembly of a heteromeric filamentous structure (Fig. 5**D-D′′**, **E-E′′**). The recruitment of proteins to cytoophidia is dependent on CTPsyn N-terminal aa 1–20 as the CTPsyn^Δ20^ aggregates are distinct from the CTPsyn filaments (Fig. 6**A-A′′**). Compared with the wild-type, western analysis detected a low amount of CTPsyn^Δ20^ protein (Fig. 4**G**, compare lane 3 with lane 2) which could be due to inefficient solubilisation of the mutant protein.

Interestingly, CTPsyn^K2E^ and CTPsyn^Y3E^ proteins which do not from filamentous structures failed to co-localise with CTPsyn – these proteins formed aggregates in the cytoplasm that were distinct from the filamentous CTPsyn cytoophidia (Fig. 6**B-B′′**, **C-C′′**). Consistently, ^pyrG/1–22^CTPsyn clusters did not assimilate into CTPsyn filaments while ^pyrG/N4K^CTPsyn co-assembled with cytoophidia (Fig. 6**D-D′′**, **E-E′′**). These results support the essential role of amino acids at position 2 (lysine) and 3 (tyrosine) in the integration of proteins to form filaments. In addition, this suggests that the N-terminal mutated CTPsyn proteins that organise into aggregates lack the capacity for homotypic and heterotypic protein-protein interactions necessary for recruitment into filaments. Indeed, these structures are not responsive to DON treatment as observed before (Fig. 3**F′, G′**). However, we cannot rule out that the mutant proteins interact with CTPsyn to form dimers or tetramers.

### 2.9 The CTPsyn^A20R^ protein shows dominant-negative activity

**Fig. 7:**
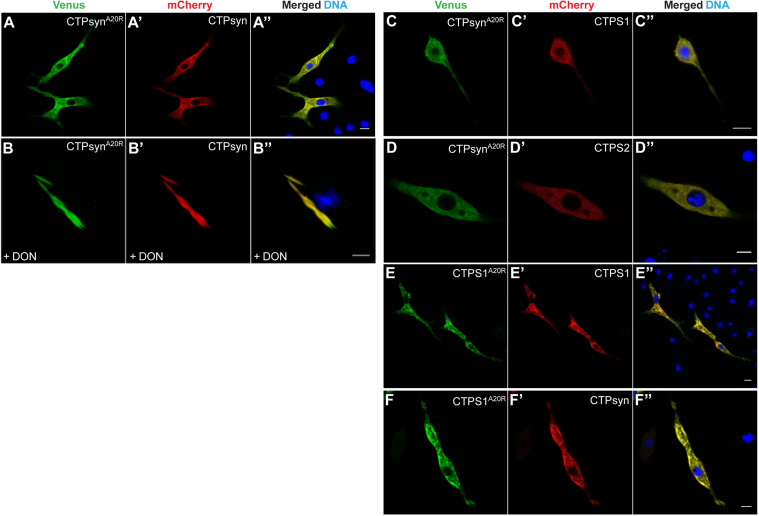
**The CTP synthase A20R mutation shows dominant-negative activity.** Co-expression of CTPsyn^A20R^-Venus (A) and CTPsyn-mCherry (A′) results in diffuse cytoplasmic distribution of both proteins (A-A′′). However, incubation of the co-expressing cells with the CTP synthase inhibitor DON restores filament formation and co-assembly of the proteins (B-B′′). Expression of *Drosophila* CTPsyn^Α20R^-Venus protein (C, D) along with mCherry-tagged CTPS1 (C′) and CTPS2 (D’) results in complete disassembly of the human cytoophidium structure leading to a uniform cytoplasmic distribution of the proteins (C′′, D′′). Similarly, the CTPS1^Α20R^-Venus protein fully dissociates the human CTPS1 cytoophidia (E-E′′) as well as *Drosophila* CTPsyn filaments (F-F′′) thus showing a potent dominant-negative activity. DNA staining with Hoescht is shown in blue and the Venus- and mCherry tagged proteins are in green and red, respectively. Scale bar=5 µm.

Co-expression of CTPsyn and CTPsyn^A20R^, however, resulted in complete disassembly of cytoophidia and intracellular foci – the overlapping fluorescent signals are distributed uniformly in the cytoplasm and did not accumulate into any cytoplasmic structures (Fig. 7**A-A′′**). Western analysis shows that the observed effect is not due to or the cause of protein degradation (Fig. 4**G**, lane 4). Thus, CTPsyn^A20R^ is capable of interacting with CTPsyn and exhibits a dominant negative function. This activity of CTPsyn^A20R^ was also observed in the presence of human CTPS1 and CTPS2 proteins which failed to polymerise (Fig. 7**C-C′′**, **D-D′′**) demonstrating an essential role of the alanine residue in determining the correct conformation of protein subunits during assembly of the higher order filamentous structure. We next investigated whether the dominant-negative effect could be observed in human CTP synthase. Indeed, human CTPS1 containing an A20R substitution (CTPS1^A20R^) could dismantle CTPS1 and CTPsyn cytoophidia fully (Fig. 7**E-E′′**, **F-F′′**). Collectively, our results reveal a structural role of the conserved alanine residue in CTPsyn necessary for its organisation into filamentous cytoophidia.

Our analysis showed that CTPsyn^A20R^ foci assembled into a filamentous structure in the presence of DON (Fig. 3**H′**). To examine if the dominant-negative activity of CTPsyn^A20R^ could be modulated by DON, we incubated cells co-expressing CTPsyn^A20R^ and CTPsyn with the drug. Indeed, addition of DON led to the formation of filamentous structures that contained both CTPsyn^A20R^ and CTPsyn proteins (Fig. 7**B-B′′**). This illustrates that binding of DON at the C-terminal domain induces a conformational change in CTPsyn that masks the filament-disassembling property of the A20R mutation, thus implying interactions between the N- and C-terminus of CTPsyn in cytoophidium formation.

### 2.10 Interaction between CTPsyn and CTPsynB disrupts filament assembly

**Fig. 8:**
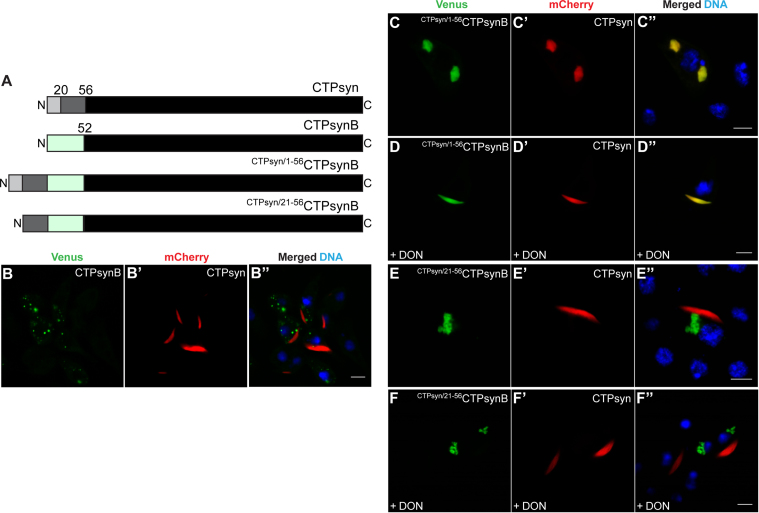
**The 20 amino acids at the CTPsyn N-terminal are required for co-assembly into cytoophidia.** (A) Schematic diagram showing CTPsyn-CTPsynB chimeric constructs wherein either aa 1–56 or aa 21–56 of CTPsyn is appended at the N-terminal of CTPsynB resulting in ^CTPsyn/1–56^CTPsynB and ^CTPsyn/21–56^CTPsynB constructs, respectively. (B-B′′) Cell expressing CTPsyn-mCherry and CTPsynB-Venus show distinct cytoplasmic distribution of both proteins. However, co-expression of Venus-tagged ^CTPsyn/1–56^CTPsynB (C) and CTPsyn-mCherry (C′) results in the formation of clusters containing both proteins (C-C′′) which organise into filaments in the presence of DON (D-D′′). ^CTPsyn/21–56^CTPsynB protein, on the other hand, forms an aggregate-like structure distinct from the CTPsyn filament (E-E′′) and is unaffected by DON treatment (F-F′′). DNA staining is shown in blue while the Venus- and mCherry-tagged proteins are in green and red, respectively. Scale bar=5 µm.

Finally, we determined if CTPsynB could be recruited to the CTPsyn filaments through N-terminal-dependent interactions. To test this, we appended aa 1–56 of CTPsyn to the N-terminal of CTPsynB resulting in the ^CTPsyn/1–56^CTPsynB construct. This modification effectively inserts 52 amino acids between the conserved N-terminal key amino acid residues (K2, Y3 and A20) and the C-terminal end of CTPsyn (Fig. 8**A**). In contrast to the cytoplasmic distribution of CTPsynB (Figs. 8**B** and 1**D**), ^CTPsyn/1–56^CTPsynB protein accumulates in distinct structures in the cytoplasm as large foci (Fig. 8**C**). This shows that the unique aa 1–56 of CTPsyn together with the C-terminal domain are sufficient for compartmentalisation of the protein into subcellular structures. The failure of ^CTPsyn/1–56^CTPsynB to assemble into linear cytoophidia may be due to increased distance between the conserved N-terminal residues and C-terminus which affects protein architecture and/or that CTPsynB-specific aa 1–52 may not favour filament assembly.

**Fig. 9:**
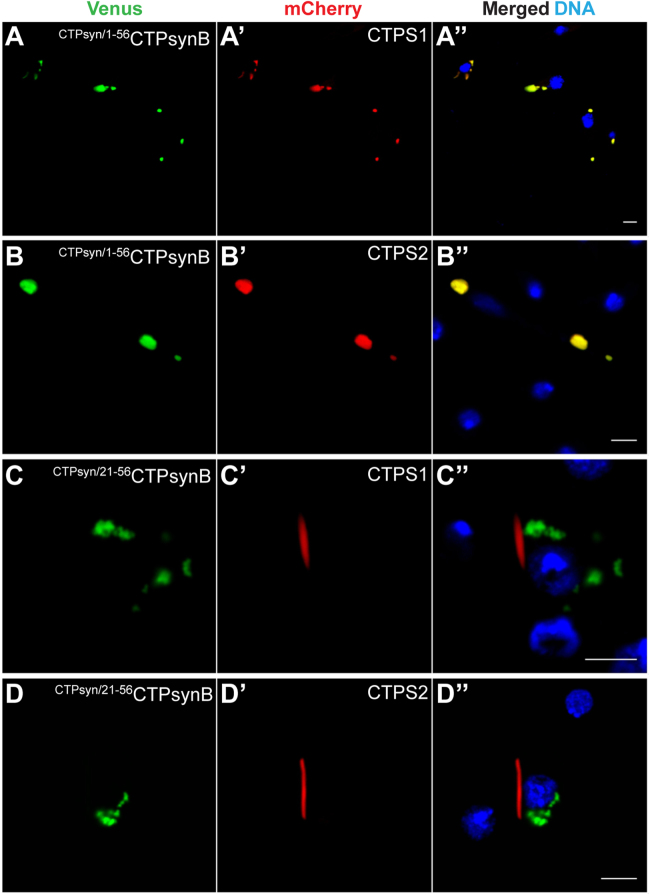
***Drosophila*****CTPsynB chimeric proteins interact with human CTP synthases in an N-terminal dependent manner.** Co-expression of the chimeric *Drosophila* CTPsynB protein containing aa 1–56 of CTPsyn (^CTPsyn/1–56^CTPsynB-Venus, A, B) and Venus-tagged human CTPS1 (A′) or CTPS2 (B′) results in significant co-localisation of the proteins in the foci (A′′, B′′) thus showing loss of CTPS1 and CTPS2 filaments. Deletion of aa 1–20 from the ^CTPsyn/1–56^CTPsynB-Venus protein (^CTPsyn/21–56^CTPsynB-Venus), however, restores CTPS1 and CTPS2 cytoophidia (C-C′′, D-D′′). Hoescht staining of DNA is shown in blue and the Venus- and mCherry tagged proteins are in green and red, respectively. Scale bar=5 µm.

Strikingly, co-expression of ^CTPsyn/1–56^CTPsynB with CTPsyn results in complete loss of cytoophidium assembly thus demonstrating a dominant-negative activity of the chimeric protein; CTPsyn protein co-localises with the ^CTPsyn/1–56^CTPsynB foci (Fig. 8**C′, C′′**). A similar effect was observed upon co-expression of ^CTPsyn/1–56^CTPsynB with the human CTPS1 and CTPS2 proteins (Fig. 9**A-A′′**, **B-B′′**). This filament-disrupting property of ^CTPsyn/1–56^CTPsynB is distinct from that of CTPsyn^A20R^ (Fig. 7**A-A′′**) and underlines the importance of proper architecture of the protein for filament formation. However, similar to CTPsyn^A20R^ (Fig. 7**B-B′′**), DON treatment resulted in the formation of a filamentous structure which contains both ^CTPsyn/1–56^CTPsynB and CTPsyn proteins (Fig. 8**D-D′′**). This phenomenon suggests that the N-terminal aa 1–56 residues together with the C-terminus are sufficient for the conformation change necessary for cytoophidium assembly while the distance between the domains is not a crucial factor for this process. Also, this shows that CTPsynB aa 1–52 do not interfere with incorporation of ^CTPsyn/1–56^CTPsynB into filaments.

Co-localisation of the proteins into foci indicates that CTPsyn is sequestered by the ^CTPsyn/1–56^CTPsynB protein possibly through N-terminal mediated protein-protein interaction. To determine this, we deleted aa 1–20 from ^CTPsyn/1–56^CTPsynB (resulting in ^CTPsyn/21–56^CTPsynB, Fig. 8**A**) and co-expressed with the wild-type CTP synthase proteins from *Drosophila* (CTPsyn) and humans (CTPS1 and CTPS2). CTP synthase now formed cytoophidia while ^CTPsyn/21–56^CTPsynB protein remained distributed as cytoplasmic aggregates (Fig. 8**E-E′′**, Fig. 9**C-C′′**, **D-D′′**) thus showing that the dominant-negative activity of ^CTPsyn/21–56^CTPsynB is mediated through N-terminal aa 1–20. Furthermore, this result demonstrates that CTPsyn N-terminal aa 1–20 are necessary and, together with the C-terminal domain, sufficient for the functional interaction that sequesters proteins into cytoplasmic structures. Consistently, in the presence of DON, ^CTPsyn/21–56^CTPsynB failed to incorporate into CTPsyn filaments (Fig. 8**F-F′′**) highlighting the critical role of N-terminal aa 1–20 in protein-protein interaction necessary for incorporation into cytoophidia.

## 3 Discussion

In this study, we investigated the role of the unique N-terminus of *Drosophila* CTP synthase in the formation of the polymeric filamentous structure. Our analyses reveal crucial roles of three conserved N-terminal amino acid residues (K2, Y3 and A20) in cytoophidium assembly. Remarkably, mutation of these amino acids result in disruption of the polymerisation process with high frequency (upto 92%, Fig. S1C) and sequestration of the corresponding protein into distinct cytoplasmic structures that differ in their responsiveness to DON (Table 1). While the identity and proximity of K2 and Y3 residues is essential for the transition of CTPsyn protein from an aggregate-like structure to linear cytoophidia, A20 plays a central role in the filamentous process and is necessary for both homo- and heteromeric filament formation, the latter revealing a dominant negative activity. Furthermore, we demonstrate heteromeric filament assembly that is dependent on N-terminal-mediated protein-protein interactions involving CTP synthase aa 1–20. Collectively, our data identify amino acids that determine the proper architecture of the protein necessary for polymerisation into filament-like structures.

*In vitro* biochemical analyses of CTP synthase in the past have revealed much about enzyme activity and its regulation. However, the identity of cis- and trans-determinants essential for the recently discovered phenomenon of filamentation remain largely obscure. Recent studies show that polymerisation of CTP synthase is favoured by CTP-mediated feedback inhibition. For example, incubation with CTP triggered filament self-assembly [6], [28] while the amino acid mutation that cripples CTP binding (E160K) dramatically reduced filament formation [32], [36]. However, the structural subunit of the filament seem to differ in prokaryotes and eukaryotes. In *E.coli* the filament consists of linearly arranged CTP synthase tetramers in a conformation that renders the enzyme inactive; mutation of the residues at the polymerisation interface abolish filament assembly [28]. On the other hand, replacement of the tetramer interface residues in the budding yeast (G148A) and *Drosophila* (G151E, R163H) protein resulted in increased filament length and frequency [32], [36]. However, substitution of amino acids constituting the dimer interface (V114F, M156I) led to shorter filaments indicating that dimers of CTP synthase likely constitute the cytoophidium [32]. As dimers are catalytically inactive, collectively, these studies indicate that inhibition of enzyme activity favours filament assembly. The crystal structure of CTP synthase shows that distinct amino acids from three subunits in the tetramer provide ATP and CTP/UTP binding surfaces thus constituting the ALase active site [10], [40], [41], [42]. Importantly, the P-loop residues (aa 15–20 in *E.coli*, aa 12–18 in humans) interact with the phosphate moieties of ATP and CTP/UTP [10], [40], [41] while the conserved lysine residue at position 18 in *E.coli* (aa 16 in human and *Drosophila*) is essential for CTP synthesis [53]. We show that the P-loop residues in *Drosophila* CTPsyn are dispensable for filament assembly (Fig. 2). Interestingly, the amino acid residues identified in this study that regulate filamentation (K2, Y3 and A20) lie in close proximity to the conserved P-loop residues. Given the architecture of the ALase catalytic site it is tempting to speculate that these residues, A20 in particular, either directly affect enzyme activity thus favouring polymerisation or induce conformation changes in the protein subunits that allows packing into a linear structure. It is possible that this region represents the primary protein interaction domain necessary for organisation of CTP synthase into a cytoophidium. Our analyses also reveal cross-talk between the ALase and GATase domains of CTPsyn in regulating cytoophidia formation. Although the GATase domain lies far away from the tetramer interfaces and is unaffected by the oligomeric state [10], [11], we show that binding of DON in this domain suppresses the filament disrupting as well as dominant negative activity of A20R mutation in the ALase domain (Figs. 3**H, H′** and 7**B-B′′**). This illustrates that local conformation changes affects quaternary structure of the protein that ultimately controls linear assembly of CTP synthase into cytoophidia. In the future, it will be interesting to investigate the higher order oliogomeric/polymeric structure(s) formed by homo- and heterotypic interactions as well as determine the enzyme activity of the mutant proteins using biochemical and biophysical approaches. Furthermore, the physiological importance and function of the cytoophidium structure *in vivo* can be studied through selective disruption of cytoophidia by CTPsyn^A20R^ protein. Live imaging analyses have shown that the long filamentous cytoophidia are formed by fusion of smaller CTP synthase foci [34]. Our results support this observation and provides evidence that filamentation of CTPsyn is a multi-step process which requires N-terminal-dependent intra- and intermolecular interaction during the later steps of the polymerisation process. Taken together, our analyses provides a framework for targeted biochemical studies and structural analyses in the future which will extend our understanding of intracellular compartmentation and structural assembly of enzymes within cells.

## 4 Conclusions

Our systematic mutational analysis of CTP synthase in *Drosophila* identifies three amino acids at the N-terminal termini that play key roles in the process of polymerisation of the enzyme to form long linear structures. We demonstrate that one of the amino acids (A20) is crucial for protein-protein interactions necessary for cytoophidia formation as its mutation results in dominant-negative activity. Remarkably the properties of these amino acids are conserved in humans CTP synthase, a drug target for anti-cancer therapy. Our study underlines the importance of proper subunit architecture and spatial relationship between amino acids residues and protein domains for filament formation. This lays the foundation for more direct biochemical and structural studies to investigate the structure-function relationship of CTP synthase in forming the fascinating intracellular structure, the cytoophidium.

## 5 Material and methods

### 5.1 Cell culture procedures

Clone 8 cells (CME W1 cl.8+) were obtained from the *Drosophila* Genomics Resource Center (DGRC) and grown in Shields and Sang M3 insect medium (Sigma) containing 2.5% fly extract, 2.5% fetal bovine serum (FBS, Sigma), 5 μg/ml insulin (Sigma), and penicillin-streptomycin (Life Technologies) in a 25 °C incubator. The cell line was verified morphologically and tested for contamination before use. For drug treatment, transfected cells were incubated with 4 μg/ml DON (Sigma) overnight as described previously [24], [32], [34]. Cells were transfected with plasmid DNA in presence of Effectene Transfection Reagent (Qiagen) as per manufacturer's instructions. Briefly, cells were seeded in a 24-well plate on coverslips and incubated overnight to ~70% confluency. For transfection, 0.2 μg of plasmid DNA was used and cells analysed by confocal microscopy three days post-transfection.

### 5.2 RNA extraction and cDNA synthesis

Total RNA was extracted from clone 8 cells and HeLa cells using miRNeasy Mini Kit (Qiagen) as per manufacturer's instructions. Genomic DNA from the samples was removed by RNase-Free DNase (Qiagen). 1 μg of RNA was used for the reverse transcription using QuantiTect Reverse Transcription Kit (Qiagen) and the cDNA was stored at −20 °C.

### 5.3 Generation of constructs

All PCR reactions were carried out in 50 μl volume using 2× Phusion Flash master mix (Thermo Fisher Scientific). The full-length coding sequences of *Drosophila* CTPsyn and CTPsynB, and human CTPS1 and CTPS2 were PCR amplified with primer pairs OL1/OL3, OL2/OL3, OL4/OL5 and OL6/OL7, respectively. The PCR products were subsequently cloned into pENTR™/D-TOPO vector (Thermo Fisher Scientific) following the user manual. To generate the truncated CTPsyn constructs, pENTR-CTPsyn plasmids were used as templates for amplification reactions using primers OL8/OL10 (CTPsyn^∆56^) and OL9/OL10 (CTPsyn^∆20^). PCR products were digested with Dpn I (NEB) at 37^0^C for 1 h, phosphorylated with polynucleotide kinase (NEB) for 30 min at 37 °C and ligated using Quick Ligation Kit (NEB) before transformation. For N-terminal swapping experiments, the CTP synthase constructs were generated by a two-fragment Gibson assembly reaction (NEB). gBlock gene fragments (IDT) coding for CTPS1^1–20^ (OL11), Ura7^1–20^ (OL12) and pyrG^1–22^ (OL13) were combined with the OL9/OL10 PCR products for the assembly reaction. Similarly, CTPsyn^1–56^CTPsynB and CTPsyn^21–56^CTPsynB encoding plasmids were constructed using gBlock gene fragments OL15 and OL16 respectively with the OL10/OL14 PCR product by Gibson assembly. To generate the point mutations, site-directed mutagenesis was carried out on pENTR-CTPsyn plasmid using primers OL17-OL52. CTPS1^A20R^ was generated on pENTR-CTPS1 and ^pyrG/T2K^CTPsyn and ^pyrG/N4K^CTPsyn were generated on pENTR-^pyrG/1–22^CTPsyn using primers OL53/OL54, OL55/OL56 and OL57/OL58, respectively. All pENTRY clones were recombined with the destination vector pAWV-Bla, pAVW, or pAW mCherry-Bla using LR clonase II (Thermo Fisher Scientific). The destination constructs were fully sequenced before using them for transfection. All primer sequences are provided in Table S1.

### 5.4 Confocal microscopy

Cells were fixed in 4% paraformaldehyde in PBS for 10 min, washed with PBT (1xPBS, 0.3% Triton X-100) twice and incubated with Hoechst 33342 (1 μg/ml) for 10 min. Endogenous CTPsyn was stained using rabbit anti-CTPsyn, antibody (Santa Cruz BioTech, sc-134457, 1:1000 dilution) and visualised using Goat anti-rabbit Alexa 488 antibody (Thermo Fisher Scientific) as described in [54]. All samples were imaged using laser-scanning confocal microscopy (Leica TCS SP5II, Leica Microsystems) using the 63x objective. At least 75 transgenic cells determined by presence of GFP and/or mCherry signal were counted for quantitation and sub-grouped into those showing a filamentous and non-filamentous structures. The image files were processed and analysed using Image J (http://imagej.net/). The lengths of cytoophidia in the cells were measured by tracing with straight or segmented lines in the Image J software.

### 5.5 Western blotting

A 2 ml culture of transiently transfected cells was harvested at 1000×g for 5 min, suspended in 150 μl of 2× SDS-sample buffer (Alfa Aesar) and boiled for 10 min. The protein samples were separated on NuPAGE 4–12% Bis-Tris gels (Thermo Fisher Scientific) along with PageRuler™ Plus Prestained Protein Ladder (Thermo Fisher Scientific) and blotted onto PVDF membrane (Amersham Hybond™) using Mini Trans-Blot transfer cell (BioRad). The primary antibodies used for staining were goat anti-GFP (Abcam, 1:500 dilution) and rabbit anti-mCherry (Abcam, 1:500 dilution) while HRP-conjugated donkey anti-goat IgG (Jackson Immunoresearch, 1:500 dilution) and donkey anti-rabbit IgG (Jackson Immunoresearch, 1:5000 dilution) were used as secondary antibodies, respectively. HRP-conjugated beta-Actin antibody (Proteintech) was used at 1:3000 dilution to visualise actin.

## Competing interests

No competing interests declared.

## Funding

This work was supported by the Medical Research Council UK. The funder has no role in study design, in collection, analysis and interpretation of data, in writing of this manuscript and decision to submit the article for publication.

## Figures and Tables

**Table 1:** Summary of intracellular structures formed by CTPsyn proteins and their modification by DON treatment.

**Protein**	**Cytoplasmic Distribution**	
	**− DON**	**+ DON**
CTPsyn	Filament	Filament
CTPsyn-Δ56	Aggregate	n.d.
CTPsyn-Δ20	Aggregate	n.d.
CTPsyn-K2E	Aggregate	Aggregate
CTPsyn-Y3E	Aggregate	Aggregate
CTPsyn-I4A	Filament	n.d.
CTPsyn-L5A	Filament	n.d.
CTPsyn-V6A	Filament	n.d.
CTPsyn-T7A	Filament	n.d.
CTPsyn-G8A	Filament	n.d.
CTPsyn-G9A	Filament	n.d.
CTPsyn-V10A	Filament	n.d.
CTPsyn-I11R	Filament	n.d.
CTPsyn-S12K	Filament	n.d.
CTPsyn-G13S	Filament	n.d.
CTPsyn-V14A	Filament	n.d.
CTPsyn-G15A	Filament	n.d.
CTPsyn-K16A	Filament	n.d.
CTPsyn-G17A	Filament	n.d.
CTPsyn-V18R	Filament	n.d.
CTPsyn-A20R	Foci	Filament
CTPsyn-A20R+CTPsyn	Diffuse	Filament
^CTPsyn/1-56^CTPsynB+CTPsyn	Large foci	Filament

n.d.=not determined

## References

[1] Liu J.L., Murphy C., Buszczak M., Clatterbuck S., Goodman R., Gall J.G. (2006). The *Drosophila melanogaster* Cajal body. J. Cell Biol..

[2] Liu J.L., Gall J.G. (2007). U bodies are cytoplasmic structures that contain uridine-rich small nuclear ribonucleoproteins and associate with P bodies. Proc. Natl. Acad. Sci. USA.

[3] Moser J.J., Fritzler M.J. (2010). Cytoplasmic ribonucleoprotein (RNP) bodies and their relationship to GW/P bodies. Int. J. Biochem. Cell Biol..

[4] Nizami Z., Deryusheva S., Gall J.G. (2010). The Cajal body and histone locus body. Cold Spring Harb. Perspect. Biol..

[5] Narayanaswamy R., Levy M., Tsechansky M., Stovall G.M., O'Connell J.D., Mirrielees J., Ellington A.D., Marcotte E.M. (2009). Widespread reorganization of metabolic enzymes into reversible assemblies upon nutrient starvation. Proc. Natl. Acad. Sci. USA.

[6] Noree C., Sato B.K., Broyer R.M., Wilhelm J.E. (2010). Identification of novel filament-forming proteins in *Saccharomyces cerevisiae* and *Drosophila melanogaster*. J. Cell Biol..

[7] O'Connell J.D., Zhao A., Ellington A.D., Marcotte E.M. (2012). Dynamic reorganization of metabolic enzymes into intracellular bodies. Annu. Rev. Cell Dev. Biol..

[8] Shen Q.J., Kassim H., Huang Y., Li H., Zhang J., Li G., Wang P.Y., Yan J., Ye F., Liu J.L. (2016). Filamentation of metabolic enzymes in *Saccharomyces cerevisiae*. J. Genet. Genom..

[9] Levitzki A., Koshland D.E. (1971). Cytidine triphosphate synthetase. Covalent Intermed. Mech. Action Biochem..

[10] Endrizzi J.A., Kim H.S., Anderson P.M., Baldwin E.P. (2004). Crystal structure of *Escherichia coli* cytidine triphosphate synthetase, a nucleotide-regulated glutamine amidotransferase/ATP-dependent amidoligase fusion protein and homologue of anticancer and antiparasitic drug targets. Biochemistry.

[11] Goto M., Omi R., Nakagawa N., Miyahara I., Hirotsu K. (2004). Crystal structures of CTP synthetase reveal ATP, UTP, and glutamine binding sites. Structure.

[12] Weng M.L., Zalkin H. (1987). Structural role for a conserved region in the CTP synthetase glutamine amide transfer domain. J. Bacteriol..

[13] Lunn F.A., Bearne S.L. (2004). Alternative substrates for wild-type and L109A *E. coli* CTP synthases: kinetic evidence for a constricted ammonia tunnel. Eur. J. Biochem..

[14] Levitzki A., Koshland D.E. (1972). Ligand-induced dimer-to-tetramer transformation in cytosine triphosphate synthetase. Biochemistry.

[15] Anderson P.M. (1983). CTP synthetase from *Escherichia coli*: an improved purification procedure and characterization of hysteretic and enzyme concentration effects on kinetic properties. Biochemistry.

[16] Pappas A., Yang W.L., Park T.S., Carman G.M. (1998). Nucleotide-dependent tetramerization of CTP synthetase from *Saccharomyces cerevisiae*. J. Biol. Chem..

[17] Kensler T.W., Cooney D.A. (1981). Chemotherapeutic inhibitors of the enzymes of the de novo pyrimidine pathway. Adv. Pharmacol. Chemother..

[18] Hatse S., De Clercq E., Balzarini J. (1999). Role of antimetabolites of purine and pyrimidine nucleotide metabolism in tumor cell differentiation. Biochem. Pharmacol..

[19] Hofer A., Steverding D., Chabes A., Brun R., Thelander L. (2001). *Trypanosoma brucei* CTP synthetase: a target for the treatment of African sleeping sickness. Proc. Natl. Acad. Sci. USA.

[20] Willoughby L.F., Schlosser T., Manning S.A., Parisot J.P., Street I.P., Richardson H.E., Humbert P.O., Brumby A.M. (2013). An in vivo large-scale chemical screening platform using *Drosophila* for anti-cancer drug discovery. Dis. Model Mech..

[21] Ingerson-Mahar M., Briegel A., Werner J.N., Jensen G.J., Gitai Z. (2010). The metabolic enzyme CTP synthase forms cytoskeletal filaments. Nat. Cell Biol..

[22] Liu J.L. (2010). Intracellular compartmentation of CTP synthase in *Drosophila*. J. Genet. Genom..

[23] Carcamo W.C., Satoh M., Kasahara H., Terada N., Hamazaki T., Chan J.Y., Yao B., Tamayo S., Covini G., von Muhlen C.A. (2011). Induction of cytoplasmic rods and rings structures by inhibition of the CTP and GTP synthetic pathway in mammalian cells. PLoS One.

[24] Chen K.N., Zhang J., Tastan O.Y., Deussen Z.A., Siswick M.Y.Y., Liu J.L. (2011). Glutamine analogs promote cytoophidium assembly in human and *Drosophila* cells. J. Genet. Genom..

[25] Liu J.L. (2011). The enigmatic cytoophidium: compartmentation of CTP synthase via filament formation. Bioessays.

[26] Aughey G.N., Liu J.L. (2016). Metabolic regulation via enzyme filamentation. Crit. Rev. Biochem. Mol. Biol..

[27] Liu J.L. (2016). The cytoophidium and its kind: filamentation and compartmentation of metabolic enzymes. Annu. Rev. Cell. Dev. Biol..

[28] Barry R.M., Bitbol A.F., Lorestani A., Charles E.J., Habrian C.H., Hansen J.M., Li H.J., Baldwin E.P., Wingreen N.S., Kollman J.M. (2014). Large-scale filament formation inhibits the activity of CTP synthetase. Elife.

[29] Shen Q.J., Kassim H., Huang Y., Li H., Zhang J., Li G., Wang P.Y., Yan J., Ye F., Liu J.L. (2016). Filamentation of metabolic enzymes in *Saccharomyces cerevisiae*. J. Genet. Genom..

[30] Buszczak M., Paterno S., Lighthouse D., Bachman J., Planck J., Owen S., Skora A.D., Nystul T.G., Ohlstein B., Allen A. (2007). The Carnegie protein trap library: a versatile tool for *Drosophila* developmental studies. Genetics.

[31] Azzam G., Liu J.L. (2013). Only one isoform of *Drosophila melanogaster* CTP synthase forms the cytoophidium. PLoS Genet..

[32] Aughey G.N., Grice S.J., Shen Q.J., Xu Y., Chang C.C., Azzam G., Wang P.Y., Freeman-Mills L., Pai L.M., Sung L.Y. (2014). Nucleotide synthesis is regulated by cytoophidium formation during neurodevelopment and adaptive metabolism. Biol. Open.

[33] Tastan O.Y., Liu J.L., Synthase Is C.T.P. (2015). Required for optic lobe homeostasis in *Drosophila*. J. Genet. Genom..

[34] Gou K.M., Chang C.C., Shen Q.J., Sung L.Y., Liu J.L. (2014). CTP synthase forms cytoophidia in the cytoplasm and nucleus. Exp. Cell Res..

[35] Zhang J., Hulme L., Liu J.L. (2014). Asymmetric inheritance of cytoophidia in *Schizosaccharomyces pombe*. Biol. Open.

[36] Noree C., Monfort E., Shiau A.K., Wilhelm J.E. (2014). Common regulatory control of CTP synthase enzyme activity and filament formation. Mol. Biol. Cell.

[37] Vetter I.R., Wittinghofer A. (1999). Nucleoside triphosphate-binding proteins: different scaffolds to achieve phosphoryl transfer. Q. Rev. Biophys..

[38] Prasad G.S. (2001). Glycine rich P-loop motif in deoxyuridine pyrophosphatase. Curr. Protein Pept. Sci..

[39] Lunn F.A., MacLeod T.J., Bearne S.L. (2008). Mutational analysis of conserved glycine residues 142, 143 and 146 reveals Gly(142) is critical for tetramerization of CTP synthase from *Escherichia coli*. Biochem. J..

[40] Endrizzi J.A., Kim H., Anderson P.M., Baldwin E.P. (2005). Mechanisms of product feedback regulation and drug resistance in cytidine triphosphate synthetases from the structure of a CTP-inhibited complex. Biochemistry.

[41] Kursula P., Flodin S., Ehn M., Hammarstrom M., Schuler H., Nordlund P., Stenmark P. (2006). Structure of the synthetase domain of human CTP synthetase, a target for anticancer therapy. Acta Crystallogr. Sect. F Struct. Biol. Cryst. Commun..

[42] Lauritsen I., Willemoes M., Jensen K.F., Johansson E., Harris P. (2011). Structure of the dimeric form of CTP synthase from *Sulfolobus solfataricus*. Acta Crystallogr. Sect. F Struct. Biol. Cryst. Commun..

[43] Park T.S., Ostrander D.B., Pappas A., Carman G.M. (1999). Identification of Ser424 as the protein kinase A phosphorylation site in CTP synthetase from *Saccharomyces cerevisiae*. Biochemistry.

[44] Choi M.G., Park T.S., Carman G.M. (2003). Phosphorylation of *Saccharomyces cerevisiae* CTP synthetase at Ser424 by protein kinases A and C regulates phosphatidylcholine synthesis by the CDP-choline pathway. J. Biol. Chem..

[45] Park T.S., O'Brien D.J., Carman G.M. (2003). Phosphorylation of CTP synthetase on Ser36, Ser330, Ser354, and Ser454 regulates the levels of CTP and phosphatidylcholine synthesis in *Saccharomyces cerevisiae*. J. Biol. Chem..

[46] Chang Y.F., Martin S.S., Baldwin E.P., Carman G.M. (2007). Phosphorylation of human CTP synthetase 1 by protein kinase C: identification of Ser(462) and Thr(455) as major sites of phosphorylation. J. Biol. Chem..

[47] Higgins M.J., Graves P.R., Graves L.M. (2007). Regulation of human cytidine triphosphate synthetase 1 by glycogen synthase kinase 3. J. Biol. Chem..

[48] Strochlic T.I., Stavrides K.P., Thomas S.V., Nicolas E., O'Reilly A.M., Peterson J.R. (2014). Ack kinase regulates CTP synthase filaments during *Drosophila* oogenesis. EMBO Rep..

[49] Wang P.Y., Lin W.C., Tsai Y.C., Cheng M.L., Lin Y.H., Tseng S.H., Chakraborty A., Pai L.M. (2015). Regulation of CTP synthase filament formation During DNA endoreplication in *Drosophila*. Genetics.

[50] Pai L.M., Wang P.Y., Lin W.C., Chakraborty A., Yeh C.T., Lin Y.H. (2016). Ubiquitination and filamentous structure of cytidine triphosphate synthase. Fly.

[51] Aughey G.N., Grice S.J., Liu J.L. (2016). The interplay between Myc and CTP synthase in *Drosophila*. PLoS Genet..

[52] Chakraborty K.P., Hurlbert R.B. (1961). Role of glutamine in the biosynthesis of cytidine nucleotides in *Escherichia coli*. Biochim. Biophys. Acta.

[53] Simard D., Hewitt K.A., Lunn F., Iyengar A., Bearne S.L. (2003). Limited proteolysis of *Escherichia coli* cytidine 5'-triphosphate synthase. Identification of residues required for CTP formation and GTP-dependent activation of glutamine hydrolysis. Eur. J. Biochem..

[54] Tastan O.Y., Liu J.L. (2015). Visualizing cytoophidia expression in *Drosophila* follicle cells via immunohistochemistry. Methods Mol. Biol..

