# Supplementary material for: Critical roles of CTP synthase N-terminal in cytoophidium assembly
Source: Exp Cell Res. 2017 May 15;354(2):122–33. doi: 10.1016/j.yexcr.2017.03.042 (PMC5405848; doi:10.1016/j.yexcr.2017.03.042)
Supplement: Supplementary file 1 — Supplementary material [file mmc1.pdf]

Supplementary Material

Critical roles of CTP synthase N-terminal in cytoophidium assembly

Yong Huang[a,b], Jin-Jun Wang[b], Sanjay Ghosh[a,1,*] and Ji-Long Liu[a,c,*]

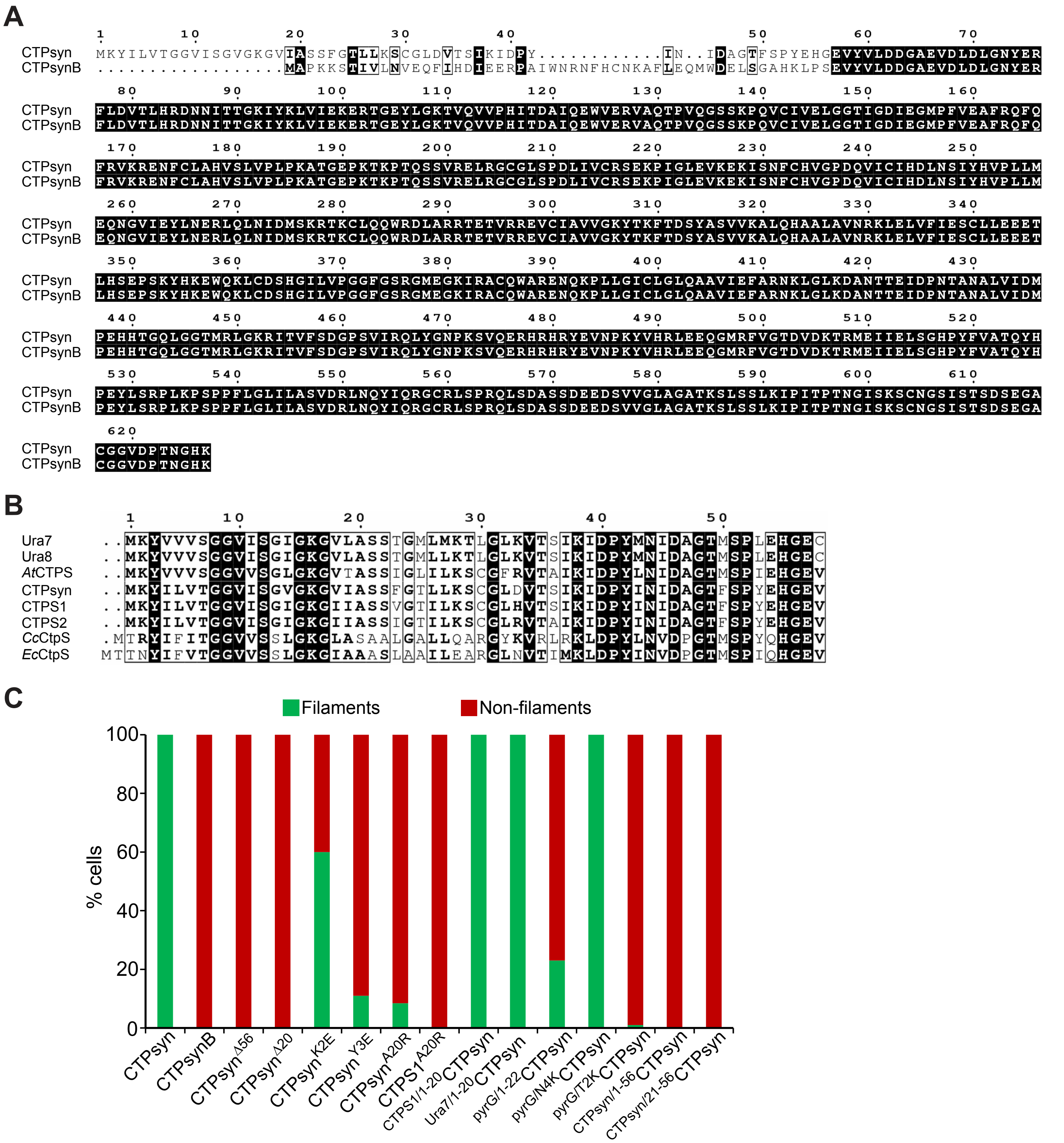

Figure S1. *Drosophila* CTPsyn has a unique N-terminal sequence that is conserved in the orthologous proteins. (A) Amino acid sequence alignment of *Drosophila* CTPsyn and CTPsynB polypeptides using the Clustal omega programme (http://www.ebi.ac.uk/Tools/msa/clustalo/) reveals a unique N-terminal for both proteins (56 amino acids for CTPsyn, 52 amino acids for CTPsynB). (B) Multiple sequence alignment of CTPsynthetase N-terminal 56 amino acids from *Drosophila melanogaster* (CTPsyn) with the corresponding CTP synthetase region from *Saccharomyces cerevisiae* (Ura7 and Ura8), *Arabidopsis thaliana* (*At*CTPS), human (CTPS1 and CTPS2), *Caulobacter crescentus* (*Cc*Ctps), and *Escherichia coli* (*Ec*Ctps) proteins. The sequence alignment was performed using Clustal omega and presented using Espirit3 (http://espript.ibcp.fr). The amino acid position shown at the top of the sequence is from the N-terminal end of the *Drosophila* protein. The identical amino acids are shaded in black. (C) Quantitation of filament-forming capacity of all proteins derived from the constructs generated in this study. Cells showing filamentous structures and clumps/aggregates (non-filaments) were counted and the percentages shown in green and red, respectively, on the y-axis of the stacked barplot. The constructs are shown on the x-axis. At least 75 Venus-positive cells were counted for each construct.

Figure S1 **Huang et al.**

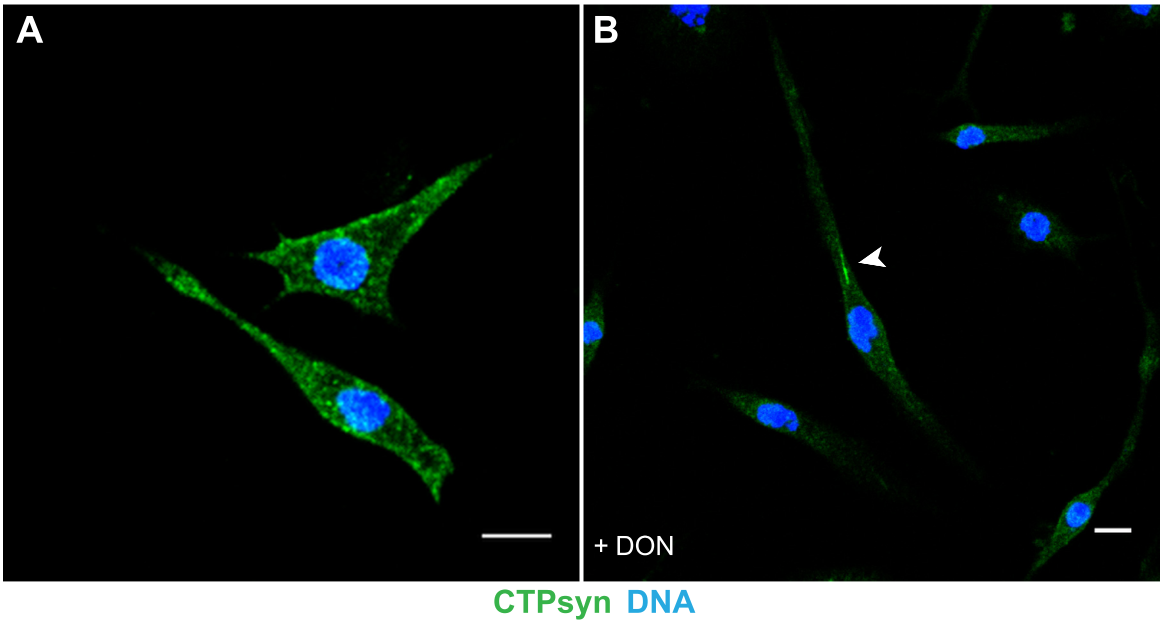

Figure S2. Distribution of CTPsyn in *Drosophila* clone 8 cells.
Immunofluoroscence staining of clone 8 cells with anti-CTPsyn antibody in absence (A) or presence of DON (B). The arrowhead shows CTPsyn filament within a cell treated with DON. Hoescht staining of DNA is shown in blue. Scale bar = 5 μm.

Figure S2

Huang et al.

Table S1. List of primers used in this study.

Oligo	Sequences (5' > 3')
OL1	CACCATGAAATACATCCTGGTAACTGGTGG
OL2	CACCATGGCGCCAAAAAAGTCCAC
OL3	CTTATGGCCATTGGTAGGATCAAC
OL4	CACCATGAAGTACATTCTGGTTACTGGTG
OL5	GTCATGATTTATTGATGGAAACTTC
OL6	CACCATGAAGTACATCCTGGTCACG
OL7	GCTTATTTCCAACTCAGCTATCC
OL8	GAGGTTTACGTTTTGGACGATGG
OL9	TCCTCGTTCGGAACGCTTTTG
OL10	CATGGTGAAGGGGGCGGC
OL11	ACAAAAAAGCAGGCTCCGCGGCCGCCCCCTTCACCA TGAAGTACATTCTGGTTACTGGTGGTGTTATATCAGG AATTGGAAAAGGAATCATTGCCTCCTCGTTCGGAAC GCTTTTGAAATCCTGTGGTCT
OL12	ACAAAAAAGCAGGCTCCGCGGCCGCCCCCTTCACCA TGAAGTACGTTGTTGTTTCAGGTGGTGTCATTTCGGG TATCGGTAAAGGTGTTCTTGCATCCTCGTTCGGAACG CTTTTGAAATCCTGTGGTCT
OL13	ACAAAAAAGCAGGCTCCGCGGCCGCCCCCTTCACCA TGACAACGAACTATATTTTTGTGACCGGCGGGGTCGT ATCCTCTCTGGGTAAAGGCATTGCCGCATCCTCGTTC GGAACGCTTTTGAAATCCTGTGGTCT
OL14	GCGCCAAAAAAGTCCACCATTG
OL15	CTTTGTACAAAAAAGCAGGCTCCGCGGCCGCCCCCTT CACCATGAAATACATCCTGGTAACTGGTGGCGTCATT AGTGGCGTGGGAAAAGGAGTGATTGCCTCCTCGTTC GGAACGCTTTTGAAATCCTGTGGTCTGGATGTAACCT CGATCAAGATTGACCCCTATATCAATATAGATGCTGG AACCTTTTCGCCTTATGAGCATGGCGCGCCAAAAAA GTCCACCATTGTGCTCAATGTGGAGCAGT
OL16	CTTTGTACAAAAAAGCAGGCTCCGCGGCCGCCCCCTT CACCATGTCCTCGTTCGGAACGCTTTTGAAATCCTGT GGTCTGGATGTAACCTCGATCAAGATTGACCCCTATA TCAATATAGATGCTGGAACCTTTTCGCCTTATGAGCA TGGCGCGCCAAAAAAGTCCACCATTGTGCTCAATGT GGAGCAGT
OL17	GCCCCCTTCACCATGGAATACATCCTGGTAAC
OL18	GTTACCAGGATGTATTCCATGGTGAAGGGGGC
OL19	CCCCCTTCACCATGAAAGAGATCCTGGTAACTGGTG
OL20	CACCAGTTACCAGGATCTCTTTCATGGTGAAGGGGG
OL21	CTTCACCATGAAATACGCCCTGGTAACTGGTGG
OL22	CCACCAGTTACCAGGGCGTATTTCATGGTGAAG
OL23	CATGAAATACATCGCGGTAACTGGTGGC

OL24	GCCACCAGTTACCGCGATGTATTTCATG
OL25	GAAATACATCCTGGCAACTGGTGGCGTC
OL26	GACGCCACCAGTTGCCAGGATGTATTTC
OL27	GAAATACATCCTGGTAGCTGGTGGCGTCATTAG
OL28	CTAATGACGCCACCAGCTACCAGGATGTATTTC
OL29	CATCCTGGTAACTGCTGGCGTCATTAGTG
OL30	CACTAATGACGCCAGCAGTTACCAGGATG
OL31	CTGGTAACTGGTGCCGTCATTAGTGGC
OL32	GCCACTAATGACGGCACCAGTTACCAG
OL33	GTAACTGGTGGCGCCATTAGTGGCGTG
OL34	CACGCCACTAATGGCGCCACCAGTTAC
OL35	GGTAACTGGTGGCGTCCGTAGTGGCGTGGGAAAAG
OL36	CTTTTCCCACGCCACTACGGACGCCACCAGTTACC
OL37	CTGGTGGCGTCATTAAAGGCGTGGGAAAAGG
OL38	CCTTTTCCCACGCCTTTAATGACGCCACCAG
OL39	GTGGCGTCATTAGTAGCGTGGGAAAAGG
OL40	CCTTTTCCCACGCTACTAATGACGCCAC
OL41	GTCATTAGTGGCGCGGGAAAAGGAGTG
OL42	CACTCCTTTTCCCGCGCCACTAATGAC
OL43	CATTAGTGGCGTGGCAAAAGGAGTGATTGC
OL44	GCAATCACTCCTTTTGCCACGCCACTAATG
OL45	CATTAGTGGCGTGGGAGCAGGAGTGATTGCCTC
OL46	GAGGCAATCACTCCTGCTCCCACGCCACTAATG
OL47	GCGTGGGAAAAGCAGTGATTGCCTCC
OL48	GGAGGCAATCACTGCTTTTCCCACGC
OL49	GTGGCGTGGGAAAAGGACGGATTGCCTCCTCGTTC
OL50	GAACGAGGAGGCAATCCGTCCTTTTCCCACGCCAC
OL51	GAAAAGGAGTGATTCGCTCCTCGTTCGGAAC
OL52	GTTCCGAACGAGGAGCGAATCACTCCTTTTC
OL53	GAAAAGGAATCATTCGCAGCAGTGTGGGCAC
OL54	GTGCCCACACTGCTGCGAATGATTCCTTTTC
OL55	GCCCCCTTCACCATGAAAACGAACTATATTTTTG
OL56	CAAAAATATAGTTCGTTTTCATGGTGAAGGGGGC
OL57	CTTCACCATGACAACGAAATATATTTTTGTGACCGGC
OL58	GCCGGTCACAAAAATATATTTCGTTGTCATGGTGAAG
